# The protective effect of isosteviol sodium on cardiac function and myocardial remodelling in transverse aortic constriction rat

**DOI:** 10.1111/jcmm.16182

**Published:** 2020-12-17

**Authors:** Qingjin Ke, Fei Liu, Yuxin Tang, Jiedi Chen, Hui Hu, Xiaoou Sun, Wen Tan

**Affiliations:** ^1^ Institute of Biomedical and Pharmaceutical Sciences Guangdong University of Technology Guangzhou China

**Keywords:** autonomic nervous system, hypertrophy, isosteviol sodium, myocardial remodelling, transverse aortic constriction

## Abstract

Pathological hypertrophy contributes to heart failure and there is not quite effective treatment to invert this process. Isosteviol has been shown to protect the heart against ischaemia‐reperfusion injury and isoproterenol‐induced cardiac hypertrophy, but its effect on pressure overload‐induced cardiac hypertrophy is still unknown. Pressure overload induced by transverse aortic constriction (TAC) causes cardiac hypertrophy in rats to mimic the pathological condition in human. This study examined the effects of isosteviol sodium (STVNa) on cardiac hypertrophy by the TAC model and cellular assays in vitro. Cardiac function test, electrocardiogram analysis and histological analysis were conducted. The effects of STVNa on calcium transient of the adult rat ventricular cells and the proliferation of neonatal rat cardiac fibroblasts were also studied in vitro. Cardiac hypertrophy was observed after 3‐week TAC while the extensive cardiac dysfunction and electronic remodelling were observed after 9‐week TAC. Both STVNa and sildenafil (positive drug) treatment reversed the two process, but STVNa appeared to be more superior in some aspects and did not change calcium transient considerably. STVNa also reversed TAC‐induced cardiac fibrosis in vivo and TGF‐β1‐induced fibroblast proliferation in vitro. Moreover, STVNa, but not sildenafil, reversed impairment of the autonomic nervous system induced by 9‐week TAC.

## 1 INTRODUCTION

Cardiac hypertrophy is a compensatory response to pressure overload and a key risk factor for heart failure.^1^, ^2^, ^3^ The cardiac function generally becomes deteriorative during the transition from compensated to decompensated cardiac hypertrophy.^4^, ^5^ The cardiac remodelling characterized by cardiac fibrosis that significantly reduces cardiac function and electrophysiological remodelling also occurs during the cardiac hypertrophy.^6^, ^7^, ^8^, ^9^ The process of cardiac hypertrophy is also accompanied by abnormal electrophysiological performance, which has a regulatory effect on cardiac isotropy.^10^, ^11^

Preventing pathological cardiac hypertrophy is a significant therapeutic goal that can postpone the progression of heart failure.^12^ The aim of current pharmacotherapies such as diuretics and angiotensin‐converting enzyme inhibitors is to reduce cardiac loading conditions or increasing cardiac contractility. Although they show some clinical benefits, improving the inotropic defect of the failing myocardium has posed some challenges.^13^ Other treatment options like sympathomimetic amines and phosphodiesterase inhibitors improve left ventricular systolic function but have a side effect of increasing the incidence of ischaemia and arrhythmias.^14^, ^15^ Similar adverse effects are associated with the use of levosimendan and omecamtiv mecarbil.^16^, ^17^ Therefore, there is a great need to develop new pharmacological agents that target relevant signalling pathways and cardiac fibrosis.^18^

Isosteviol is a beyerane diterpene obtained from acid hydrolysis of stevioside. It is widely known for its sweet taste and effects on the cardiovascular system in traditional medicines of South America.^19^ Several studies have reported that isosteviol possesses a cardioprotective effect in ischaemia‐reperfusion heart injuries, protects the heart against isoproterenol‐induced cardiac hypertrophy and reduces arrhythmia.^20^, ^21^, ^22^, ^23^, ^24^ However, whether isosteviol has a protective effect on pressure overload‐induced pathological cardiac hypertrophy has not yet been investigated. STVNa is the sodium salt of isosteviol and has better water solubility than isosteviol.^25^ In our study, we investigated whether STVNa can decrease cardiac fibrosis, improve cardiac function and reverse autonomic nervous system (ANS) balance in TAC‐induced hypertrophy rats and the potential mechanisms.

## 2 MATERIALS AND METHOD

### 2.1 Experimental animals

Nine‐week‐old male Wistar rats, weighing 180‐220 g (Certification No. 44008500011386, SPF grade), were purchased from the Experimental Animal Center of Sun Yat‐Sen University (Guangzhou, China). The rats were maintained on a standard rodent chow diet with 12‐hour light and dark cycles. The experimental procedures were carried as per the guidelines of the National Institute of Health Guide for the Care and Use of Laboratory Animals from the National Institute of Health.

### 2.2 Animal models

Constriction of the transverse thoracic aorta was performed to induce pressure overload for 3 weeks (3‐week TAC) or 9 weeks (9‐week TAC) as previously described.^26^ Briefly, each rat was anaesthetized under 3% pentobarbital sodium (IP) followed by intubation and ventilation on a fixed‐volume positive‐pressure respirator (Model 683, Harvard Apparatus). Midline sternotomy was performed and the aorta visualized. A 4‐0 suture was placed between the brachiocephalic artery and the left common carotid artery. The suture was tightened around a blunt 21‐gauge needle placed adjacent to the aorta. The needle was then removed and the chest closed. Approximately 5% of the rats in our study died after TAC. The sham group underwent the same operation but without aortic constriction. After the surgery, the rats were housed in an individual cage with a standard rodent diet. This study was approved the Institutional Animal Care and Research Advisory Committee of the Institute of Biomedical & Pharmaceutical Sciences at Sun Yat‐Sen University (permit number 20140515171141; approved 15 May 2014).

### 2.3 Experimental protocol

Two days after the surgery, the drug or vehicle was administrated by gavage. Experiment groups included control group (TAC + vehicle), low‐dose (TAC + STV (L), 1 mg/kg/d), middle‐dose (TAC + STV (M), 2 mg/kg/d) and high‐dose (TAC + STV (H), 8 mg/kg/d) STVNa groups. Sildenafil (TAC + Sil, 70 mg/kg/d) was used as a positive control group. After 3 or 9 weeks, haemodynamic parameters and electrocardiograph of the rats were measured and monitored, respectively. Finally, pentobarbital sodium was used to euthanatize the rats and tissue collected to analyse the change in histology.

### 2.4 Surgical preparation

After 3 or 9 weeks, the rats were anaesthetized, intubated and ventilated as previously described. The conversion of the catheter signal unit was performed according to the manufacturer's instructions.^27^ The femoral artery was catheterized to monitor systemic arterial pressure. The rats were placed on a homeothermic blanket to maintain their body temperatures at 37°C. A middle‐neck incision was made, the parotid glands moved aside, and a thin muscle layer around the throat bluntly dissected to expose and isolate the right carotid artery. One suture was knotted at the distal end of the artery, the other suture inserted beneath the carotid artery, and a clip placed at the proximal end of the artery. The middle suture was made of a very loose knot, then a 1.9‐F conductance Pressure‐Volume (P‐V) catheter (Model SPR‐838, Millar Instruments Inc) was catheterized through a tiny incision. It was quickly advanced into the left ventricle along the long axis until a stable PV loop signal was obtained.

### 2.5 Haemodynamic measurements

To derive systolic function indices that were less influenced by loading conditions and cardiac mass left ventricular, P‐V relations were measured by transiently compressing the inferior vena cava under the diaphragm with a silicone tube‐tipped forceps. Mechanical ventilation was suspended, followed by a transient inferior vena cava occlusion (<5 seconds) to reduce left ventricular preload, and approximately 20 cardiac cycles were selected. The data were obtained in 5‐second intervals. The changes of peripheral resistance were also examined by a polyethylene arterial catheter (PE10) connected to a pressure transducer inserted into the distal abdominal aorta via the femoral artery retrograde. To determine the influence of muscle conductance to ventricle volume, 40 μL hypertonic saline (30%) was injected into the right jugular vein to obtain parallel conductance values. All the data were acquired using the PowerLab system (PL16/35, AD Instruments).

### 2.6 ECG analysis

The electrocardiogram (ECG) was measured by the II Einthoven lead using the PowerLab system. Ten minutes' record of ECG was digitally acquired at 2 kHz before any experiment.^28^ Heart rate variability spectral analysis was performed using a fast Fourier transformation. Oscillatory components were separated into very low‐frequency (VLF, approximately 0.04 Hz), low‐frequency (LF, 0.04‐0.6 Hz) and high‐frequency (HF, 0.6‐2.5 Hz) bands.^29^, ^30^ Heart rate variance (HRV) components were expressed in normalized units (n. u.) as a percentage of total power, less the very low‐frequency component. Efferent parasympathetic vagal nerve activity is a major contributor to the high‐frequency component while the low‐frequency component results from both sympathetic and vagal influences. The low/high‐frequency ratio is commonly utilized as a representation of sympathovagal balance.

### 2.7 Histology study

Tissue sections of hearts of the rats were fixed by 10% neutral‐buffered formalin, embedded in paraffin, cut into 3 μm serial sections and stained with Haematoxylin and Eosin (H&E), Picrosirius red or phalloidin. The digital images were captured by a microscope system (DM4000‐E1, Leica). Picrosirius Red (PSR) staining was used to grade the extent of fibrosis and the amount of F‐actin was assessed by phalloidin staining. We measured the cross‐sectional area of cells and interstitial collagen fraction using Image‐Pro Plus software (Media Cybernetics). Four or five different hearts were selected for the analysis of each group.

### 2.8 Ca^2+^ transient analysis of ventricular muscle cells

Adult rat ventricular cells were isolated by standard methods^31^, ^32^ and incubated in Tyrode's solution for 2 hours as previously described.^33^ Then the cells were incubated with 1 μmol/L Fluo‐4AM (F‐14217, Invitrogen) at room temperature for 30 minutes and washed by Tyrode's solution which contained 1.0 mmol/L Ca^2+^ to remove excess dye. Ca^2+^ transients were elicited through a pair of platinum electrodes by field stimulation (pulse time 5 ms, frequency 1 Hz and voltage 5‐10 v). Cells were stimulated at 1 Hz to produce steady‐state conditions. The confocal line scan imaging was performed by the confocal microscope system (LSM 710, Zeiss). Fluorescence was excited at 488 nm and measured at >520 nm. The scan was set at a line scan pixels of 300 × 1, a speed of about 300~400 μs/line, and a total number of scans of 30 000. Ca^2+^ transients were obtained by measuring the time/fluorescence intensity changes from the corresponding line scan images. After recording Ca^2+^ transients of myocytes in the blank, 1 μmol/L STVNa was added into the Ca^2+^‐containing Tyrode's solution, incubated for 2 minutes and then Ca^2+^ transients recorded again.

### 2.9 MTT assay of cardiac fibroblasts

Neonatal rat cardiac fibroblasts were isolated as described previously.^34^ Briefly, hearts from newborn 1/2‐day‐old Sprague‐Dawley were minced on ice, and cells isolated by trypsinization incubation at 37°C. Non‐cardiomyocytes were separated from the cardiomyocytes by differential attachment rates. This step allowed for the preferential attachment of fibroblasts to the bottom of the culture dish. Nonadherent or weakly adherent cells were removed, fresh medium added and the adherent cells allowed to grow until confluence. The cells were passaged every 2 to 3 days using 0.05% trypsin‐EDTA solution and grown at 37°C in 5% CO_2_ in DEME/F_12_ medium with 5% foetal calf serum. The viability of cardiac fibroblast was assessed by the 3‐(4,5‐dimethylthiazol‐2‐yl)‐2, 5‐diphenyltetrazolium bromide (MTT) assay on 96‐well plates.^35^ After 24 hours of culture, 0.5 mg/mL MTT substrate was added, and the cells incubated for an additional 4 hours. They were then solubilized with DMSO for 10 minutes at room temperature. Absorbance was measured at 490 nm.

### 2.10 Statistical analysis

All data were presented as mean ± SEM. Differences between multiple groups were compared by analysis of variance (ANOVA) followed by Tukey's multiple comparisons test. The two‐group analysis was performed by t test (paired or unpaired as appropriate). All *P*‐values were 2‐sided and a significance level of *P* < .05 set.

## 3 RESULT

### 3.1 The effects of STVNa on TAC‐induced cardiac hypertrophy

**Figure 1:**
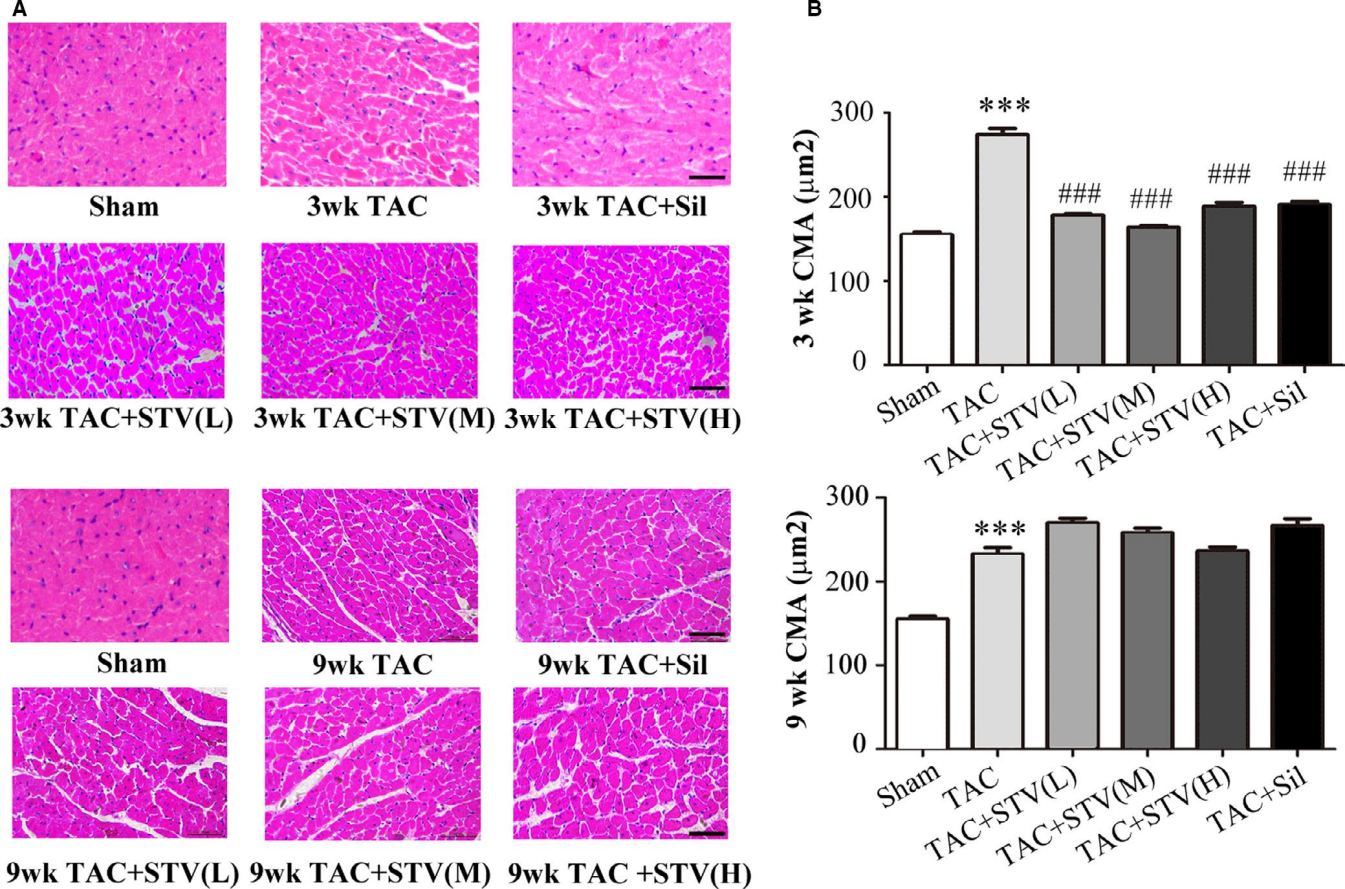
STVNa amelioration of cardiac myocyte hypertrophy. A, Representative left ventricular myocardial sections stained with Haematoxylin and Eosin; B, The data of cardiomyocyte area (CMA) in the sham group, TAC (transverse aortic constriction) group, and STV (L, M, H doses) (STVNa treatment) or Sil (sildenafil treatment) groups both for 3 wk (n = 5) and 9 wk (n = 5), wk, week. Data are mean ± SEM. ***P < .001 vs sham group, ^###^ *P* < .001 vs TAC group

Adult Wistar rats were subjected to TAC for 3 or 9 weeks and treated with vehicle, STVNa and sildenafil. There was a significant increase in heart to body weight ratio (HW/BW) in the 3‐week TAC group and the 9‐week TAC group by 34.6% and 14.8%, respectively (Table 1). This was accompanied by 76.4% and 49.6% increase of cardiomyocyte cross‐sectional area 3‐week TAC group and the 9‐week TAC group, respectively (Figure 1A,B). Treatment with STVNa could obviously decreased the LW/BW ratio in the 3‐week TAC groups and the cardiomyocyte cross‐sectional area tended to decrease in the left ventricle. The hearts of the 9‐week TAC group rats were heavier than those of the 3‐week group. However, HW/BW of the 9‐week TAC group did not increase compared to the 3‐week TAC group due to the 21.9% concurrent increase in body weight.

**Table 1:** Heart weight to body weight characteristics of the study groups

	Sham	TAC	TAC + STV(L)	TAC + STV(M)	TAC + STV(H)	TAC + Sil
3 wk						
HW (g)	0.68 ± 0.03	0.91 ± 0.06	0.72 ± 0.03	0.78 ± 0.05	0.83 ± 0.04	0.76 ± 0.04
BW (g)	272.6 ± 10.82	270.75 ± 8.41	250.5 ± 5.17	258 ± 2.89	284.5 ± 14.4	264.5 ± 7.9
HW/BW (mg/g)	2.497 ± 0.101	3.361 ± 0.155**	2.862 ± 0.099#	3.008 ± 0.162	2.908 ± 0.096#	2.86 ± 0.117#
9 wk						
HW (g)	0.68 ± 0.03	0.94 ± 0.04	0.98 ± 0.06	0.88 ± 0.06	0.98 ± 0.08	0.96 ± 0.08
BW (g)	272.6 ± 10.82	330.2 ± 16.3	338.2 ± 12.83	312.8 ± 19.21	339 ± 22.12	313.8 ± 5.73
HW/BW (mg/g)	2.497 ± 0.101	2.866 ± 0.079*	2.936 ± 0.275	2.817 ± 0.133	2.89 ± 0.162	3.053 ± 0.267

Note

Values are mean ± SEM; n = 5 rats. TAC, Transverse aortic constriction; Sham, underwent the same operation, but without aortic constriction; TAC + STV(L), TAC treated with STVNa (1 mg/kg/d); TAC + STV(M), TAC treated with STVNa (2 mg/kg/d); TAC + STV(H), TAC treated with STVNa (8 mg/kg/d); TAC + Sil, TAC treated with sildenafil (70 mg/kg/d). HW, heart weight measured in g; BW, body weight measured in g; HW/BW, heart weight to body weight ratio (mg/g); wk, week.

* *P* < .05.

** *P* < .01 vs sham.

^#^ *P* < .05 vs TAC.

### 3.2 STVNa prevents TAC‐induced cardiac dysfunction

**Figure 2:**
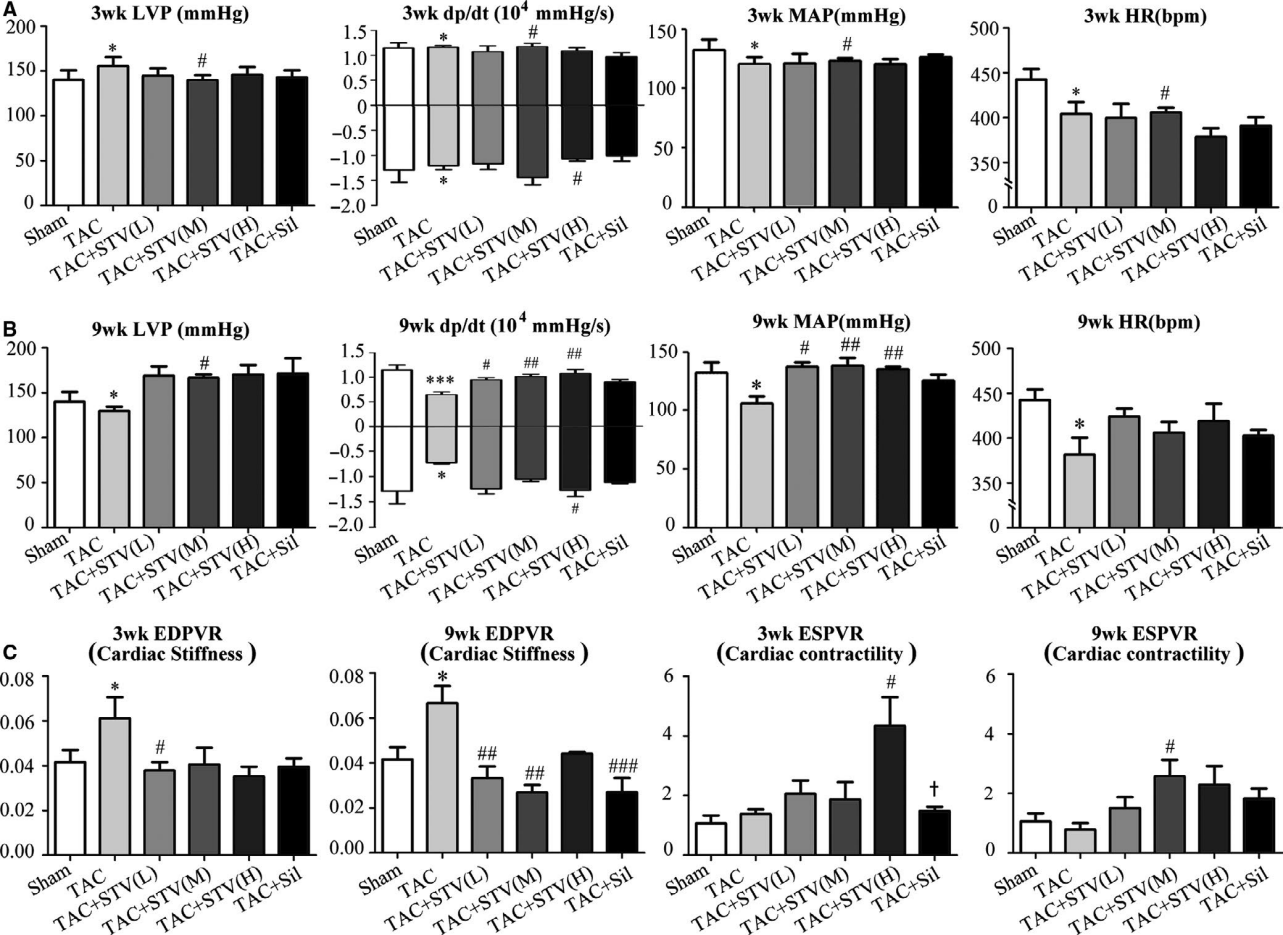
In vivo cardiac function improvement by STVNa treatment. A, Summary data for LVP, left ventricular pressure; dp/dt, peak rate of pressure decline (diastolic indexes) and pressure ascend (systolic indexes); MAP, mean arterial pressure; HR, heart rate in 3‐wk groups (A) and 9‐wk groups (B). Pressure‐volume relationship measured by preload reduction (C). EDPVR, the slope of end‐diastolic pressure‐volume relationship represents cardiac stiffness and ESPVR, the slope of end‐systolic pressure‐volume relationship represents end‐systolic elastics. STVNa decreased cardiac stiffness induced by TAC and increased cardiac contractility, but sildenafil did not have significant effect on cardiac contractility. *P < .05 ***P < .001 vs sham group; ^#^ *P* < .05, ^##^ *P* < .01, ^###^ *P* < .001 vs TAC group, ^+^ *P* < .05 vs TAC + STV (H) group, n = 5

Cardiac function, evaluated by left ventricular pressure (LVP), dp/dt_max/min_, mean artery pressure (MAP), and heart rate (HR) were not significantly different between the 3‐week TAC group and the sham control group as shown in Figure 2A. In the 9‐week TAC control group, dp/dt_max/min_, MAP and HR values decreased significantly compared to the sham group (*P* < .05), which were reversed by STVNa or sildenafil treatment. STVNa in 8 mg/kg/d showed a better protective effect than sildenafil (Figure 2B).

The results also showed that EDPVR increased by 47.2% in the 3‐week TAC control group, and reached a significant diversity by an increase of 60.4% in the 9‐week TAC control group compared to the sham group (*P* < .05). The STVNa treatment, especially in a dose of 8 mg/kg/d, led to a significant increase in ESPVR in both 3‐ and 9‐week TAC groups. This observation suggested that STVNa could improve cardiac contractility independent of hypertrophic heart damage. However, sildenafil treatment did not show the same effect (Figure 2C).

### 3.3 The effects of STVNa on electrophysiological alterations

**Figure 3:**
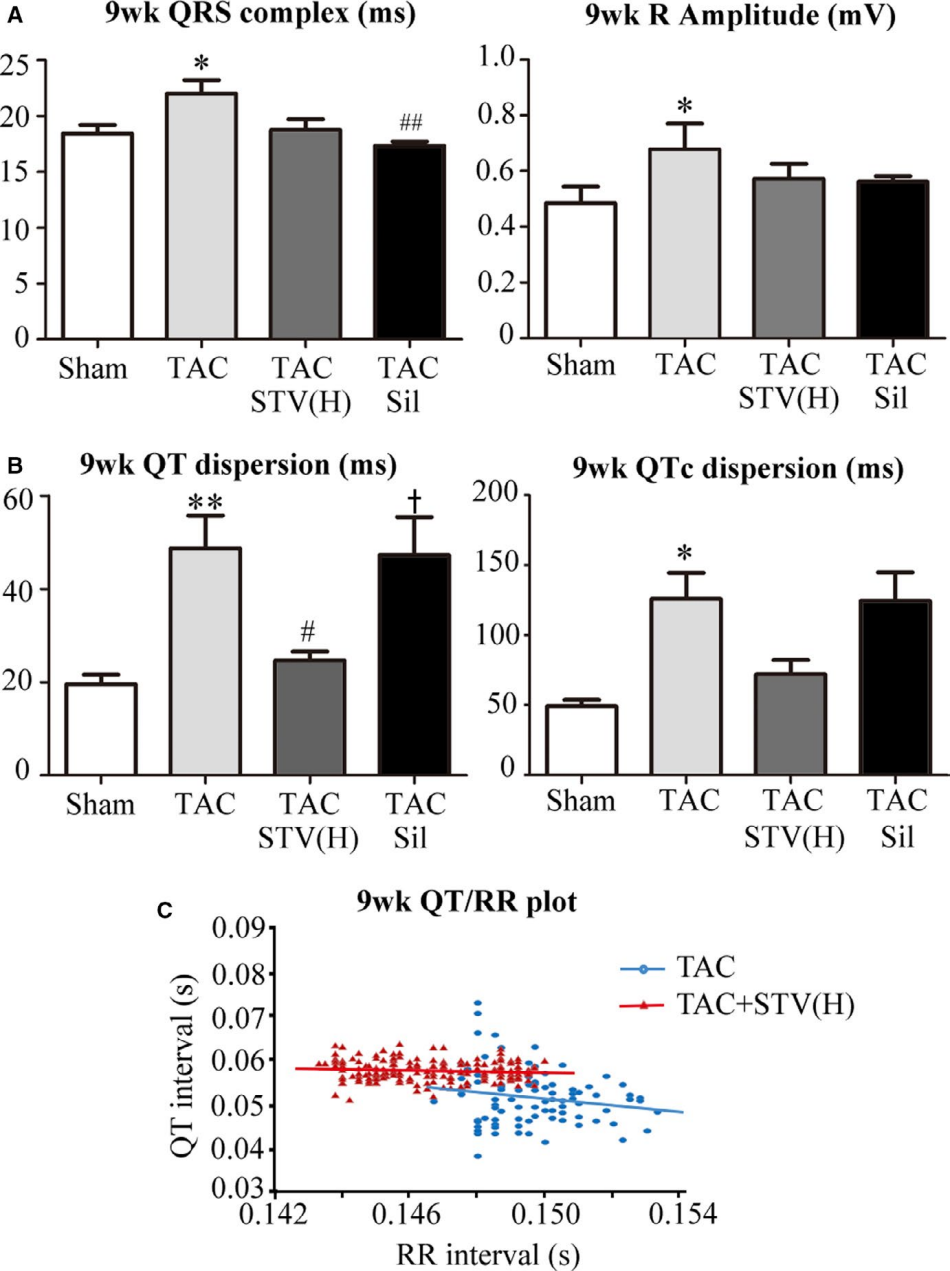
STVNa reversing the alteration of cardiac electric cardiograms. A, QRS interval measured in ms, R amplitude measured in mV. TAC for 9 wk induced an increase of QRS duration and R amplitude which was reversed by STVNa and sildenafil treatment. B, QT and QTc dispersions measured in ms High QT dispersion indicates a high risk of cardiac arrhythmia and is reduced by STVNa but not sildenafil treatment. C, Representative QT/RR plot in the TAC group (blue) and TAC treated with STVNa high dose (red). In 9‐wk groups, QTd increased in the TAC group and could be reversed by STVNa treatment. *P < .05, **P < .01 vs sham group; ^#^ *P* < .05, ^##^ *P* < .01 vs TAC group, ^+^ *P* < .05 vs TAC + STV group, n = 5

Cardiac electrical remodelling is generally accompanied by the cardiac hypertrophy, so we studied the effects of STVNa on electrophysiological alterations in the hypertrophic heart. Compared with the sham control group, rats exposed to TAC for 9 weeks had a long QRS duration and high R amplitude on both STVNa and sildenafil treatment as shown in Figure 3A. The QT (*P* < .01) and QTc (*P* < .05) dispersions also increased significantly after 9 weeks of TAC‐induced surgery, which could be reversed by STVNa treatment but not sildenafil treatment (Figure 3B). Representative QT/RR plot reflecting the relationship between repolarization and rates showed an increased propensity for bradycardia‐dependent ventricular arrhythmia after 9‐week TAC and a risk reduction by STVNa treatment (Figure 3C).

### 3.4 STVNa improves cardiac function without increasing cardiac energy demand

**Figure 4:**
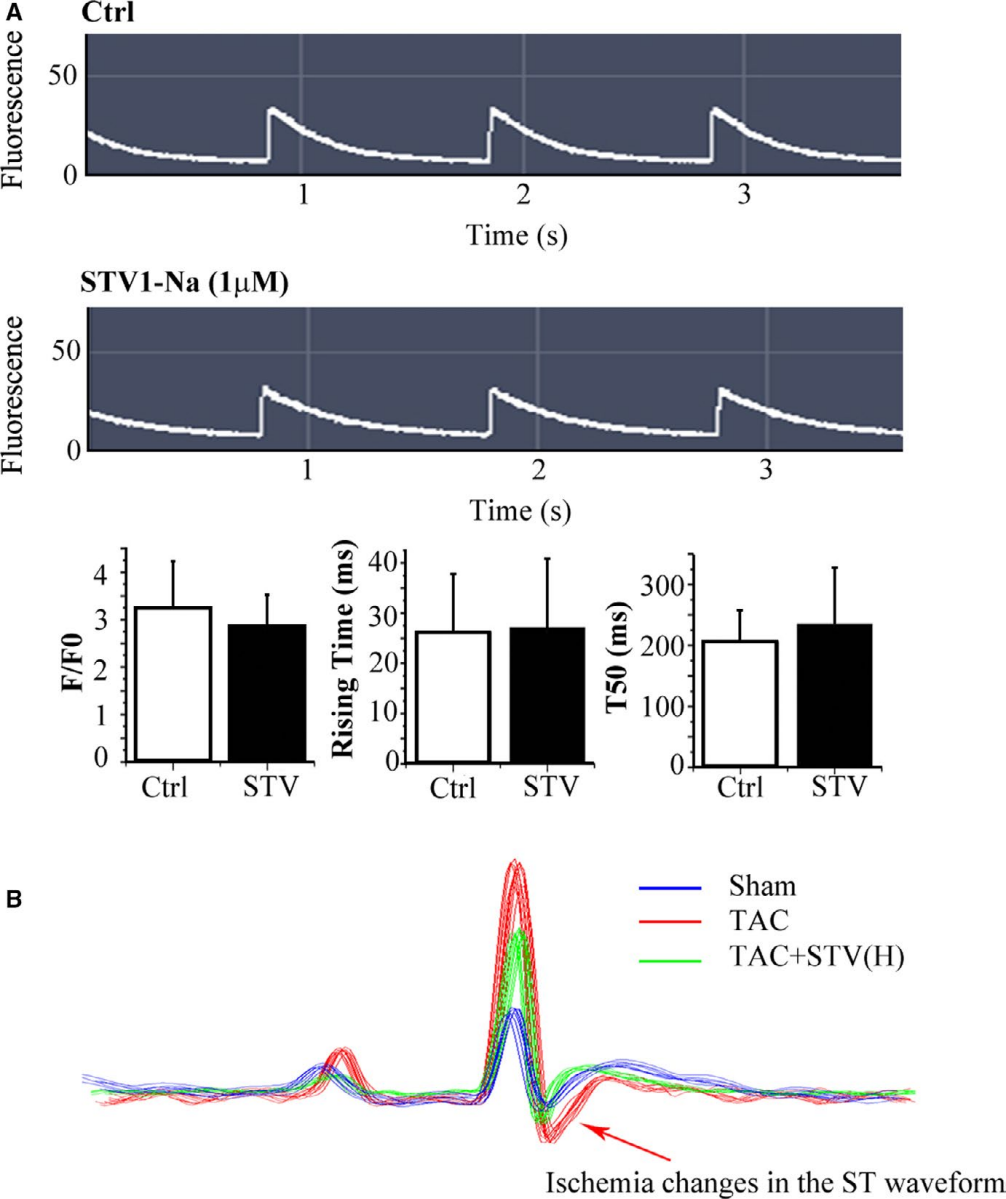
STVNa treatment did not increase oxygen consumption. A, Representative image of calcium transient, a fluo‐4 AM (1 μmol/L) loaded acutely on isolated rat myocardial cells, indicating that STVNa (1 mmol/L) improved cardiac function without increasing intracellular calcium level. The bottom bar graph shows the summary data for intracellular calcium level and the rate of decay of the intracellular calcium transient measured by F/F0, The time and T50 were increased according to calcium fluorescence images. Ctrl, control cells; STV, STVNa‐treated cells; n = 3. B, Representative ECG recording in sham (blue), TAC (red) and STVNa (high‐dose group) treatment group (green)

To test whether STVNa increases energy demand as compensation for improvement in LV systolic function, we analysed intracellular Ca^2+^ level and ST waveform variation in ECG. We examined intracellular Ca^2+^ in freshly isolated Fluo‐4‐loaded ventricular myocytes. There was no significant difference in the Ca^2+^ transient amplitude before and after adding 1 μmol/L STVNa, and the RD50 (decay to 50% amplitude) did not change significantly (Figure 4A). The ST waveform downward of ECG tracing showed that TAC‐induced hypoxic change, which was recovered with STVNa treatment as shown in Figure 4B.

### 3.5 The inhibition effects of STVNa on actin remodelling and fibrosis formation

**Figure 5:**
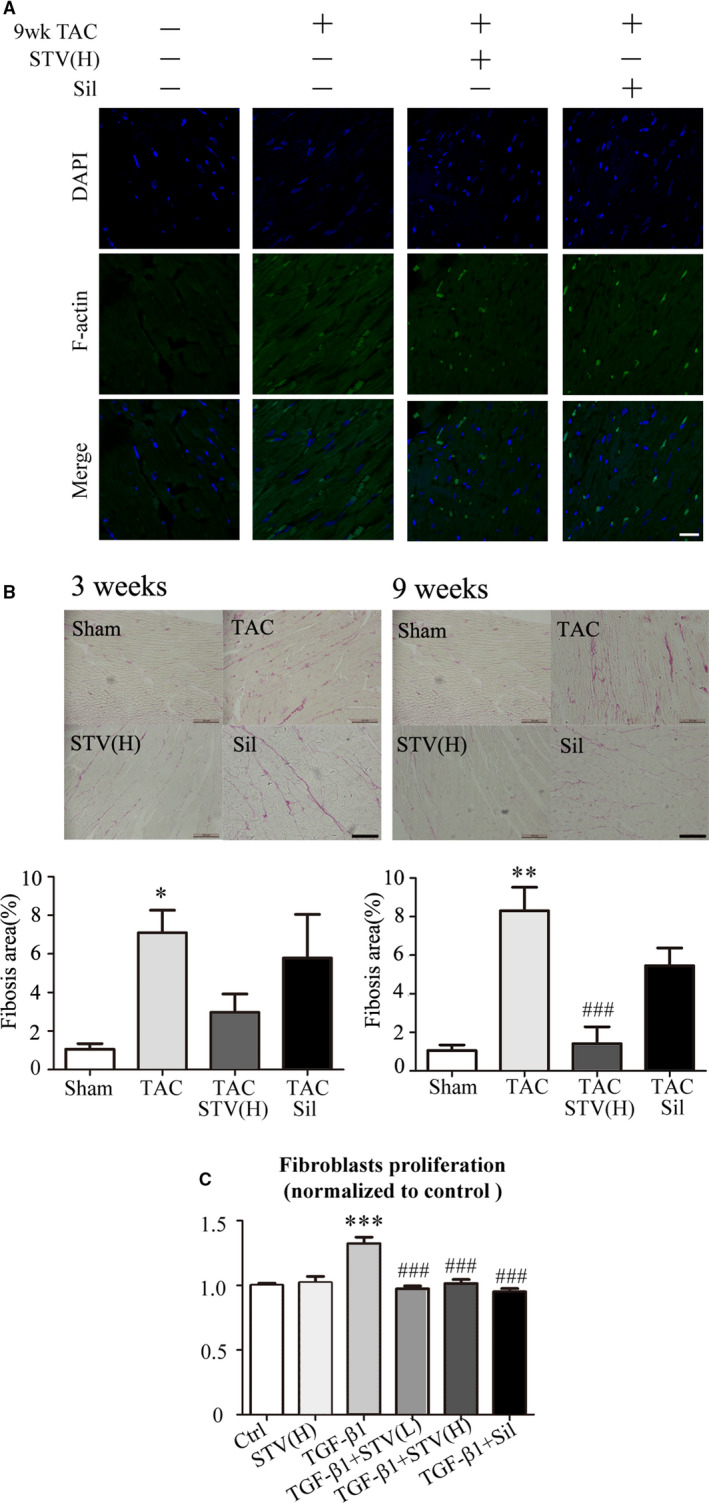
STVNa prevents cardiac fibrosis induced by pressure overload. A, Representative F‐actin‐stained photomicrographs (×630, oil). Ventricular cross‐sections were stained with FITC‐conjugated phalloidin (green). B, Fibrosis measured in ventricular cross‐sections stained with Picrosirius Red stain (red) 3 and 9 wk after TAC surgery. Scale bar = 100 mm, n = 5. C, STVNa inhibition of the TGF‐β1‐stimulated fibrotic response in isolated neonatal rat cardiac fibroblasts. *P < .05, **P < .01, ***P < .001 vs sham group or control; ^###^ *P* < .001 vs TAC group or TGF‐β1‐treated group

The level of F‐actin increased after TAC for 9 weeks and was reversed by STVNa (8 mg/kg/d) or sildenafil (70 mg/kg/d) treatment (Figure 5A). Compared to the sham control group, the collagen content also increased by 5.7‐ and 7.5‐fold in 3‐ and 9‐week TAC control groups respectively. When treated with 8 mg/kg/d STVNa, the interstitial fibrosis reduced by 58.2% and 80.8% in 3‐ and 9‐week TAC groups, respectively as shown in Figure 5B. Sildenafil exhibited less inhibition effect on cardiac fibrosis compared to STVNa.

We further investigated the potential mechanism underlying the anti‐fibrotic effects of STVNa in vitro. The results showed that TGF‐β1 (5 ng/mL) significantly increased the proliferation of neonatal rat cardiac fibroblasts, which could be blocked by STVNa (1 µmol/L or 10 µmol/L) or sildenafil (100 µmol/L) (Figure 5C).

### 3.6 STVNa stables the autonomic nervous system which impaired by TAC

**Figure 6:**
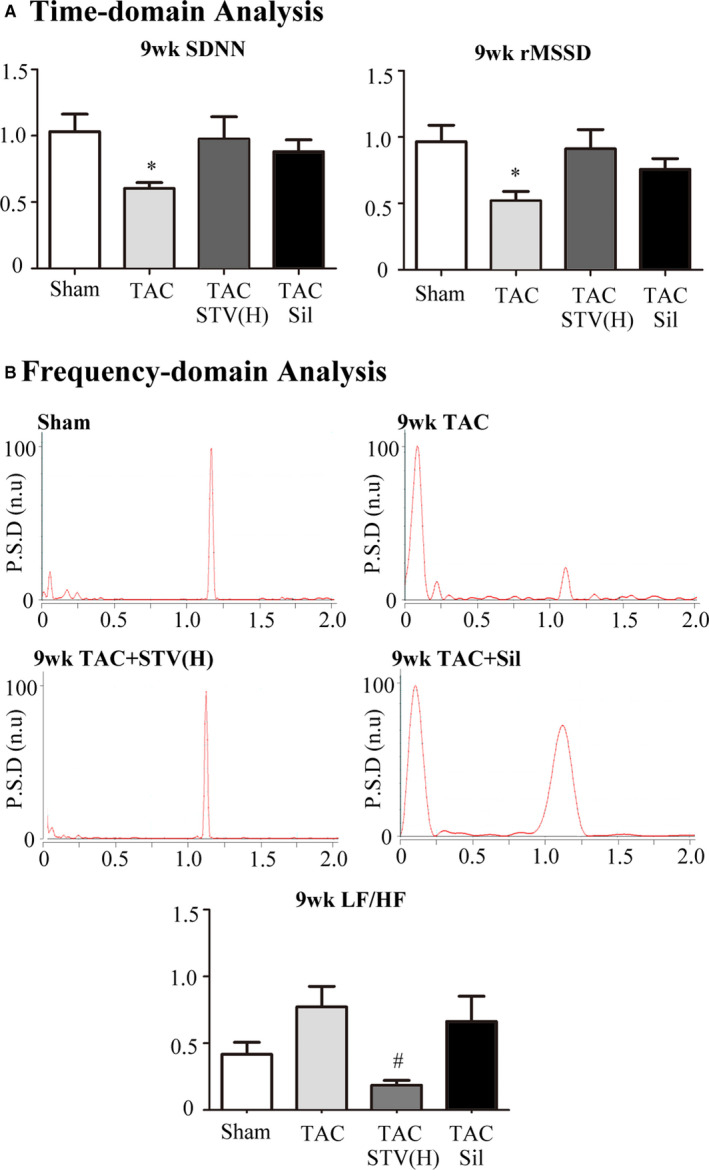
HRV analysis of aortic constriction rats with or without drug treatment. A, Group data of SDNN (standard derivation of RR interval) measured in ms and rMSSD value for sham, TAC, TAC treated with STVNa (TAC + STV) and TAC‐treated sildenafil (TAC + Sil) groups. B, Representative traces of the power spectral density of HRV in the sham group, 9‐wk TAC group, and TAC with STVNa or sildenafil treatment groups. PSD, Power spectral density expressed in normalized units (n. u.). *P < .05 vs sham group; ^#^ *P* < .05 vs TAC group

At 9 weeks, TAC rats presented significantly lower values (*P* < .05) of SDNN (standard derivation of RR interval) and rMSSD (root mean square of successive differences), which indicated lower vagal nerve activity in the 9‐week TAC group. As shown in Figure 6A, both STVNa and sildenafil treatment increased SDNN and rMSSD values. Figure 6B shows representative power spectrum analysis of RR variability. Rats exposed to TAC for 9 weeks displayed marked changes in the distribution of the relative spectral components of HRV with a significantly higher LF/HF ratio compared to sham controls (*P* < .05), which was reversed by STVNa but not Sildenafil treatment. It appeared that STVNa had also a better effect on improving the ANS homeostasis than sildenafil.

## 4 DISCUSSION

The present study found that STVNa could significantly inhibit myocardial hypertrophy after 3 weeks of TAC. It could also improve cardiac function without increasing energy requirement or cytosolic Ca^2+^. Moreover, it decreased the vulnerability to arrhythmias after 9 weeks of TAC. STVNa achieves its cardioprotective effects probably through the inhibition of cardiac fibrosis in vivo and in vitro. Our findings also showed that STVNa was superior to sildenafil in the cardioprotective effects such as the regulation of ANS homeostasis.

The actin cytoskeleton structure disorders are a well‐characterized histological change in TAC rats which can promote vascular smooth muscle hypertrophy.^36^ In our study, STVNa treatment reversed the unbalanced polarization of actin between monomer and polymer induced by TAC. Pressure overload exerts mechanical stress on the ventricles and can trigger myocyte hypertrophy and interstitial fibrosis.^37^ Increased cardiac collagen deposition is an important character of interstitial fibrosis.^38^ In the present study, TAC induced a time‐dependent increase in myocardial interstitial fibrosis. The STVNa treatment reduced the formation of collagen to increase myocardial compliance and contractility. This inhibition effect of STVNa was better than the continued treatment of sildenafil. The TGF‐1 signalling pathway plays a critical role in myocardial fibrosis following pressure overload, mediating collagen production.^37^, ^39^ Our results showed that STVNa prevented TGF‐1‐induced proliferation of neonatal rat cardiac fibroblasts.

The interstitial fibrosis not only increases myocardium stiffness but also poses undesirable electrophysiological effects by disrupting the normal electrical connectivity of cardiac tissue that enhances the vulnerability to arrhythmias.^40^ Left ventricular hypertrophy increases the risk of cardiac events and arrhythmia.^41^ Previous studies have shown that left ventricular hypertrophy increases the QT and QTc dispersions.^42^ Prolonging of the QRS complex causes diffuse myocardial damage through the replacement of myocardium by fibrosis.^43^ In this study, the results showed that QT dispersion, QTc dispersion and QRS duration as well as R‐wave amplitude in the 9‐week TAC group were larger than those treated with STVNa which could be attributed to the increase in collagen deposition. Treatment with STVNa was more effective than sildenafil in electrocardiogram analysis, which was mainly due to the reduction in the formation of collagen. This indicated that myocardial fibrosis slows conduction by interposing collagen bundles between strands of myocytes.

Cardiac hypertrophy is a complicated dynamic process that affects the regulation of the ANS. ANS function disorder, especially the vagus nerve dysfunction is closely associated with cardiovascular disease in the development and prognosis.^44^, ^45^ In our study, we found the value of SDNN and rMSSD could reflect better the functional status of the cardiac vagus nerve. Besides, the LF/HF ratio serves as an indicator of sympathovagal balance.^46^, ^47^ We observed a high imbalance in sympathetic modulation in hypertrophy rats. These findings suggested that cardiac autonomic functions are impaired by pressure overload and that STVNa treatment can reduce such changes hence reducing the risk of arrhythmia and sudden death.

Currently, the main therapy for heart failure is positive inotropic agents. However, they increase the cardiac myocyte intracellular calcium, which in turn induces a series of adverse effects.^48^ Another inotropic agent, levosimendan, is a calcium sensitizer that can improve cardiac function without increasing oxygen demand or intracellular calcium concentration.^49^ Nonetheless, it also has some adverse effects such as hypotension, atrial and ventricular arrhythmias, and possibly early mortality as observed in a REVIVE study.^16^ Omecamtiv mecarbil, a cardiac myosin activator, improves heart contraction and stroke volume without affecting oxygen consumption^50^ but shows a dose‐related increase in systolic ejection time resulting in myocardial ischaemia in high dose.^17^ Sildenafil, a phosphodiesterase‐5A inhibitor, had been shown to significantly improve in vivo heart function in mice exposed to chronic pressure overload but does not fully reverse the hypertrophy and myocardial remodelling.^51^ Similarly, we observed that sildenafil did not reverse the electrophysiology of TAC rats and had no effect on the stabling autonomic nervous system. This implied that sildenafil was unable to decrease the risk of arrhythmia and mortality. STVNa has been reported many pharmacological activities in our previous study. For instance, Isosteviol ameliorates diabetic cardiomyopathy in rats by inhibiting ERK signalling pathways and inhibit the production of reactive oxygen species (ROS) and mitochondrial membrane potential (MMP).^22^, ^23^ In addition, our previous studies also demonstrated that STVNa significantly inhibited H9c2 cell and rat primary cardiomyocyte cell surface, restored MMP and morphological integrity, and decreased the expression of mitochondrial function‐related proteins fission 1 and dynamin‐related protein 1.^24^ Therefore, from what has been discussed above, STVNa may be more targets for drugs. On the other hand, STVNa improved the heart function by increasing cardiac contractility and decreasing cardiac stiffness without changing intracellular Ca^2+^ concentration or increasing oxygen requirement. It also inhibited interstitial collagen fibre formation. This suggested that STVNa has dual function on both myocardial function and cardiac fibrosis, and therefore, there could be another mechanism, through which it carries its function, that still needs to be studied.

## 5 CONCLUSION

The results of the present study suggest that STVNa reverses the marked hypertrophy and cardiac fibroblast proliferation induced by TAC. Moreover, STVNa improves the cardiac function without increasing Ca^2+^ level or oxygen requirement in cardiomyocyte, protects the heart from arrhythmia and maintains electrophysiological stability. All in all, STVNa has great potential to be used for the treatment of hypertrophy and cardiac fibrosis diseases.

## CONFLICT OF INTEREST

The authors declare no conflict of interest.

## AUTHOR CONTRIBUTION

**Qingjin Ke:** Writing‐original draft (equal). **Fei Liu:** Formal analysis (equal); Visualization (equal). **Yuxin Tang:** Data curation (equal). **Jiedi Chen:** Data curation (equal). **Hui Hu:** Formal analysis (equal); Visualization (equal). **Xiaoou Sun:** Conceptualization (equal); Project administration (equal); Writing‐review & editing (lead). **Wen Tan:** Conceptualization (equal); Funding acquisition (equal); Project administration (equal).

## Data Availability

The data that support the findings of this study are available on request from the corresponding author. The data are not publicly available due to privacy or ethical restrictions.
